# Beyond carbonate biomineralization: why prokaryote-driven CO_2_ sequestration demands holistic evaluation

**DOI:** 10.3389/fbioe.2025.1690042

**Published:** 2025-11-17

**Authors:** Richard Schinteie, Veena Nagaraj, Linda Stalker, Nai Tran-Dinh, David J. Midgley

**Affiliations:** 1 CSIRO Energy, Lindfield, NSW, Australia; 2 Indian Ocean Marine Centre, Crawley, WA, Australia; 3 CSIRO Energy, Kensington, WA, Australia; 4 CSIRO Energy, Pullenvale, QLD, Australia; 5 Centre for Bioinnovation, University of the Sunshine Coast, Sippy Downs, QLD, Australia

**Keywords:** microbially induced carbonate precipitation (MICP), carbon dioxide (CO_2_), carbon sequestration, biomineralization, respiration, metabolism, carbonate

## Abstract

Microbially induced carbonate precipitation (MICP) offers a promising biological approach to sequester atmospheric CO_2_ as stable mineral carbonates, mitigating climate change impacts. This perspective highlights the complexity underpinning prokaryote-driven biomineralization processes, emphasizing the necessity for holistic evaluation beyond simple carbonate formation. Key metabolic pathways such as carbonic anhydrase-mediated CO_2_ hydration, ureolysis, photosynthesis, and sulfate reduction contribute variably to mineral precipitation and the carbon footprint. Furthermore, calcium carbonate polymorphs with varying stability forms can affect carbon storage durability, while net carbon sequestration estimates often overlook critical factors including respiratory CO_2_ release, growth phases, and embodied emissions in microbial nutrient substrates. Finally, differentiating between transient microbial organic carbon and long-term mineral carbon storage is essential for accurate carbon accounting. Lifecycle carbon footprints vary significantly with metabolic strategies and substrate choices, impacting sustainable application prospects. Advancing MICP as an effective carbon removal technology requires integrated assessment of microbial physiology, environmental interactions, and process lifecycle emissions to optimize CO_2_ drawdown with environmental and economic viability.

## Introduction

The escalating challenges of climate change to a warming planet highlight the critical need for a diverse array of carbon removal technologies (Schweitzer et al., 2021), with microbially induced carbonate precipitation (MICP) increasingly recognized as a promising candidate (e.g., Mitchell et al., 2010; Okyay and Rodrigues, 2015; Gilmour et al., 2024; Wilcox et al., 2025). MICP harnesses the metabolic versatility of prokaryotes to drive the precipitation of stable carbonate minerals, notably calcium carbonate (CaCO_3_), thereby locking away atmospheric CO_2_ into solid form (e.g., Zhu and Dittrich, 2016).

A range of biotic and abiotic factors have been shown to contribute to MICP and often act in combination to achieve biomineralization. Central to many biological processes is the enzyme carbonic anhydrase, which catalyzes the hydration of CO_2_ to form bicarbonate (e.g., Meldrum and Roughton, 1933; Douglas and Beveridge, 1998; Smith and Ferry, 2000; Fu et al., 2021). Other common metabolic strategies relevant to MICP include various ammonia-producing strategies (e.g., via urease or deamination of amino acids during catabolism) as these elevate pH (e.g., Kamennaya et al., 2012; Clarà Saracho and Marek, 2024), photosynthetic uptake of CO_2_ to shift carbonate equilibrium (e.g., Riding, 2006), and dissimilatory sulfate reduction (e.g., Lin et al., 2018; Castro-Alonso et al., 2019) where H_2_S production is the mechanism by which pH is increased. Additionally, microbial cell walls and extracellular polymeric substances (EPS) present in biofilms, further assist by trapping divalent cations and providing additional nucleation surfaces, ultimately promoting or modulating the formation and stability of carbonate minerals (e.g., Zhu and Dittrich, 2016). The rate of prokaryote-driven carbonate precipitation is dependent on pH, availability of nucleation sites, concentration of dissolved inorganic carbon (DIC) and saturation of divalent Ca^2+^ and Mg^2+^ ions (e.g., Castanier et al., 2000; Zhu and Dittrich, 2016).

Advocates of CO_2_ sequestration via MICP emphasize its wide-ranging potential for stable carbon storage, citing laboratory successes where bacterial activity accelerated carbonate mineralization rates by orders of magnitude (e.g., Power et al., 2016; Abdelsamad et al., 2022). Unlike many conventional carbon sequestration methods, MICP can proceed under ambient conditions, reducing energy inputs and offering the promise of long-term CO_2_ storage through the formation of stable minerals (e.g., Wilcox et al., 2025).

Despite these promising attributes, much of the current literature does not fully consider limitations. First, whole-system carbon accounting is often incomplete. Second, the physiological constraints of the microorganisms themselves — respiration, growth phase and tolerance to elevated CO_2_ — are insufficiently understood in MICP systems. Third, the geochemical stability of the precipitated carbonates is highly dependent on the mineral type, local pH and environmental conditions, raising concerns about the potential for carbonate dissolution and the re-release of sequestered CO_2_.

**FIGURE 1:**
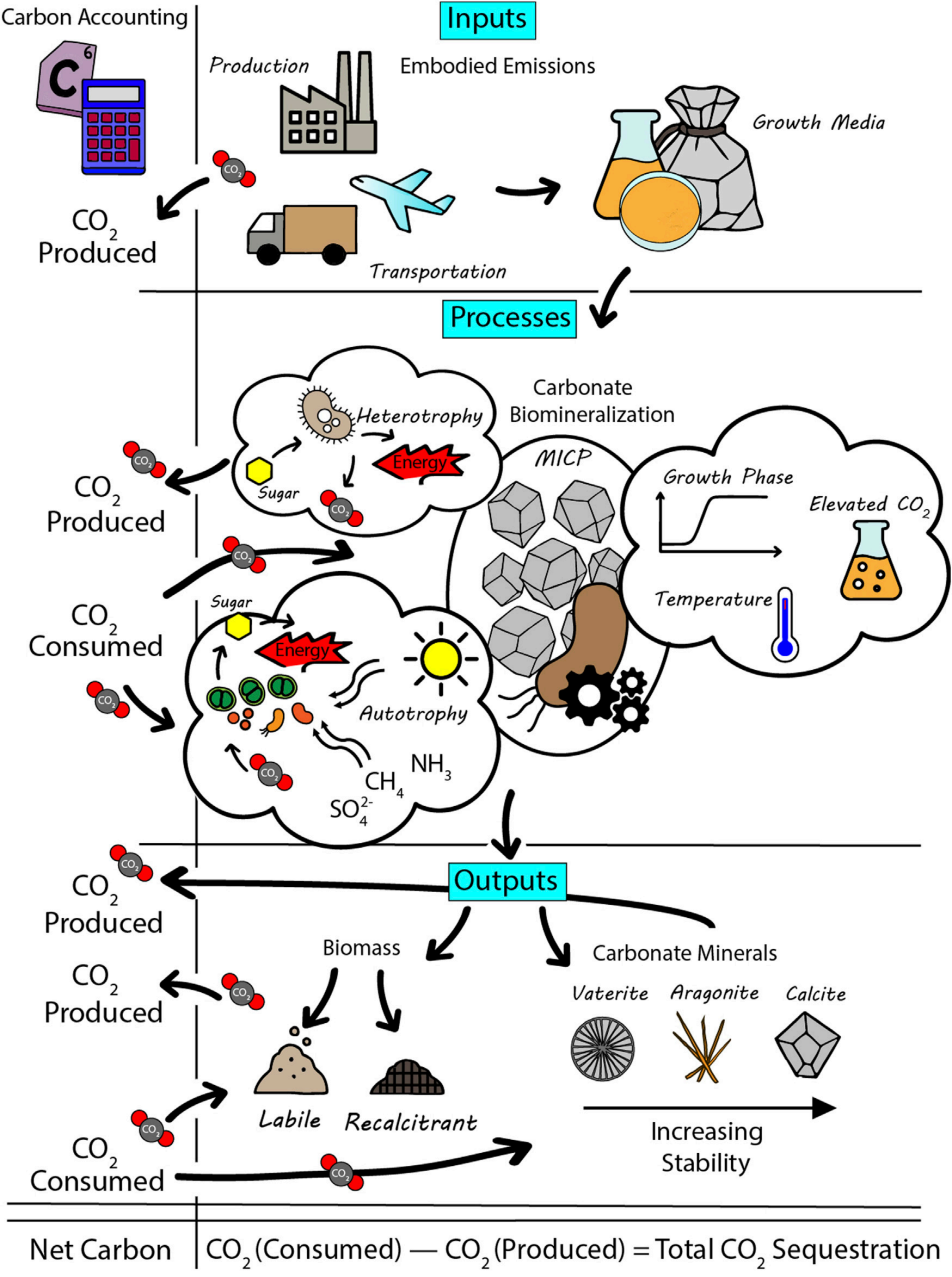
Integrated overview of carbon flows in microbially induced carbonate precipitation (MICP) systems, highlighting the full carbon accounting from inputs to outputs.

The above-mentioned issues are not only critical for scientific accuracy but also for informing policy, monitoring, verification and accounting (MVA) costs and carbon trading frameworks. Carbon markets and regulatory bodies increasingly demand evidence of permanence in sequestration projects (e.g., Meitner, 2024), requiring mechanisms like MICP that offer long-term storage to be heavily scrutinised. These issues are explored in this perspective (Figure 1) with the aim of providing a pathway to progress these technologies out of laboratories and into broader use. Table 1 provides a concise summary of the key quantitative data discussed throughout this article.

**TABLE 1:** Summary of key published quantitative parameters related to microbially induced carbonate precipitation (MICP) processes and associated carbon footprints.

Category/Parameter	Quantitative Value(s)	Notes	Source
Yeast extract CO_2_e emissions	3.34 kg CO_2_e/kg	71% processing, 23% agricultural sourcing; remainder transport/packaging	CarbonCloud 2025; Hagman et al., 2014; Vásquez Castro et al., 2023
Urea CO_2_ emissions	1.8 kg CO_2_/kg	Produced via Haber-Bosch process	Smith et al., 2020; Luo et al., 2023
Laboratory vs. industrial grade chemicals	∼18%–49.6% emission reduction	Replacing laboratory-grade with industrial-grade calcium chloride	Porter et al. (2021)
By-product nutrient sourcing	Higher emission reduction	Using industrial by-products as nutrients	Porter et al. (2021)
MICP-heterotrophic CaCO_3_ footprint	∼2.06–3.91 kg CO_2_/kg CaCO_3_	Highest, due to energy-intensive inputs	Porter et al. (2021)
MICP-autotrophic CaCO_3_ footprint	∼1.2 kg CO_2_/kg CaCO_3_	Lower direct emissions	Porter et al. (2021)
Carbonic anhydrase-only CaCO_3_	∼0.67 kg CO_2_/kg CaCO_3_	Lowest reported for MICP	Porter et al. (2021)
Microbial carbon use efficiency (CUE)	0.53 ± 0.25 (mean ± Standard Error)	Fraction lost to respiration, mean across prokaryotes	Saifuddin et al. (2019)
Pure glucose, aerobic CUE	∼0.6	Typical yield with pure cultures, glucose, and O_2_	Geyer et al. (2016)
High-CUE microbes	Up to ∼0.9	Exceptionally efficient metabolic strains	Saifuddin et al. (2019)
CO_2_ inhibition of growth	Up to 40% CO_2_	Bacterial growth linearly inhibited up to this concentration	Eklund (1984)
Optimal MICP temperature for commonly studied organisms	25–30 °C	For *Sporosarcina pasteurii*; most studies use mesophilic temperatures	Omoregie et al. (2017)
Precipitation, stationary phase	>24 h	*Sporosarcina silvestris*: active mineralization during constant stationary phase	Seidel et al. (2025)

The table includes carbon dioxide emissions for various substrates such as yeast extract and urea, metabolic carbon use efficiency (CUE), inhibition effects of elevated CO_2_ on microbial growth, and optimal experimental conditions reported in recent studies. Values are cited from multiple sources as detailed in the manuscript and provide a consolidated reference for metabolic, environmental, and operational factors influencing MICP efficacy and sustainability.

## Carbon accounting

The apparent simplicity of MICP as a carbon sequestration strategy belies a relatively complex carbon footprint. A critical oversight in current literature is the frequent absence of a holistic understanding of carbon balances, which requires evaluating all carbon inputs and outputs across the entire process lifecycle. This omission risks significantly overestimating net CO_2_ sequestration by ignoring critical emissions associated with processes such as nutrient production, metabolism, and other operational conditions. Currently, existing studies rarely validate CO_2_ sequestration when accounting for these factors, often failing to provide explicit, quantitative carbon footprint values for essential substrates like yeast extract or glucose that would be needed for the cultivation of MICP-associated microbes at an industrial scale.

### Embodied emissions in materials for MICP

Recent industrial life-cycle assessments have begun quantifying these substrate-related emissions in carbon dioxide equivalent, or CO_2_e, expressing the total greenhouse gas emissions as an amount of CO_2_ equivalent. For example, producing 1 kg of yeast extract powder in the European Union generates 3.34 kg of CO_2_e emissions, with processing accounting for 71% of the total, agricultural sourcing making up 23%, and transport and packaging comprising the remaining portion (CarbonCloud, 2025). Indeed, factors such as the production and sourcing of nutrients for yeast cultivation, the energy required to maintain optimal growth conditions, subsequent processing steps, and losses through respiration and fermentation all contribute to making yeast extract a relatively high CO_2_ emitter (Hagman et al., 2014; Vásquez Castro et al., 2023). Urea, which is often cited as an important component of some MICP approaches, is a highly energy-intensive product, emitting approximately 1.8 kg of CO_2_ per kg of urea primarily due to fossil fuel consumption during the Haber-Bosch process (e.g., Smith et al., 2020; Luo et al., 2023). The use of urea together with yeast extract would therefore compound the emission profile of any MICP process conducted on such media. To better understand how changes in input materials affect emissions in MICP, readers are directed to Porter et al. (2021), who highlight that carefully selecting and optimizing input sources can significantly lower emissions. Notably, they found that replacing laboratory-grade calcium chloride with industrial-grade alternatives reduced emissions by ∼18%–49.62%. Even greater reductions were observed when industrial by-products were used as nutrient sources. Other options, such as making a judicious choice of less carbon-intensive nitrogen sources, can help reduce the overall environmental impact of microbial cultivation. Further work to quantify and subsequently reduce embodied emissions would be of significant assistance in progressing these technologies in terms of economics and sustainability.

### MICP metabolic processes affect emissions

The rate of MICP can vary depending on which metabolic pathway predominates, environmental factors, and microbial activity levels (e.g., Fahimizadeh et al., 2022). Likewise, the carbon footprint of MICP also varies depending on the specific biochemical process employed. According to the life cycle assessment by Porter et al. (2021), heterotrophic processes, such as ureolysis, are the most carbon-intensive, producing 2.06–3.91 kg CO_2_ per kilogram of precipitated calcium carbonate. This high footprint is primarily attributed to the use of energy-intensive inputs like purified urea. By contrast, autotrophic processes generate lower direct emissions, ranging from ∼1.2 kg CO_2_ per kilogram of CaCO_3_. Notably, the use of only the carbonic anhydrase enzyme results in the lowest carbon footprint, at ∼0.67 kg CO_2_ per kilogram of CaCO_3_. However, the relatively high monetary costs of purified enzymes can be prohibitive for use in many industries (e.g., Jo et al., 2013).

### Biomass vs. mineral material for carbon locking

Microbial communities, especially autotrophs, drive carbon draw down both via MICP and the formation of biomolecules. The latter ranges in the degree of permanence from labile (LOM) to refractory organic matter, each with different stability profiles (e.g., Dranseike et al., 2025). LOM, produced through microbial carbon assimilation, is unstable and typically returns to the atmosphere within years via respiration and decomposition (e.g., Visser et al., 2016; Hoikkala et al., 2016), limiting its sequestration potential. In contrast, mineral carbonates can provide long-term carbon storage, emphasizing the importance of distinguishing between these forms in carbon accounting. This duality is especially evident for CO_2_ uptake by cyanobacteria, where the balance between organic matter formation and carbonate mineralization varies by species and environment (e.g., Kamennaya et al., 2012; Jung et al., 2024). Therefore, robust accounting methods are essential, as failing to distinguish between different forms of sequestered carbon can result in a significant overestimation of net CO_2_ removal. Mineral carbonates are typically measured using mineralogical analyses (e.g., X-ray diffractometry, scanning electron microscopy), while transient LOM requires dynamic methods such as isotopic tracers (δ^13^C) and decomposition studies (e.g., Preston et al., 2006). Without clear differentiation, net CO_2_ removal can be overestimated if transient LOM is credited as stable mineral carbon. Measurements of total inorganic carbon (TIC) *versus* total organic carbon (TOC) can be used to distinguish and quantify sequestration by analysing the difference between the two forms of carbon (e.g., Jones et al., 2023).

## Other physiological considerations

### Respiration

Microbial respiration represents a key process in MICP, yet this factor receives limited attention in most experimental studies for CO_2_ sequestration. Many investigations emphasize the ability of microbes to convert carbon into mineral forms, often neglecting comprehensive accounting of respiration within the same system.

Respiration always results in less carbon loss than the total carbon input, since net growth cannot occur if all consumed carbon is respired. The relationship between carbon consumption and retention in microbial biomass is described by carbon use efficiency (CUE; e.g., Geyer et al., 2016; Mendonça et al., 2024). For heterotrophic microbes, this metric expresses the proportion of carbon retained in biomass relative to total carbon consumed, while for autotrophic organisms, CUE is defined as the ratio of carbon retained in biomass to carbon fixed. For example, a heterotrophic microbe with a CUE of 0.5 loses approximately half of consumed carbon to respiration, whereas a CUE of 0.8 indicates a more efficient mode of growth, with only 20% of carbon lost to respiration.

Variation in CUE among prokaryotes is substantial (Saifuddin et al., 2019), making it essential to determine CUE values for target microbial strains used in MICP applications. Saifuddin et al. (2019) indicate that, on average, about half of consumed carbon (mean = 0.53 ± 0.25 Standard Error) is lost to respiration across prokaryotes, though considerable variability exists among species. Closely related taxa tend to exhibit broadly similar CUE values, yet some species demonstrate notably higher efficiency. In addition, variations in CUE reflect differences in energy conservation and biomass formation efficiency, which are influenced by both the nature of the substrate and the organism’s metabolic capabilities.

Under aerobic conditions with ample glucose availability, pure cultures often exhibit a yield of approximately ∼0.6 (Geyer et al., 2016). In certain cases, particularly when utilizing more reduced substrates or when the organisms display exceptionally efficient metabolic performance, yields can approach ∼0.9 (Saifuddin et al. 2019). Therefore, selection of substrates and microbial strains with elevated CUE values may enhance the overall efficiency of MICP processes.

### Growth phase

Most MICP studies are performed entirely during the exponential growth phase, when microbial populations are rapidly increasing. It is during this period that both calcium carbonate precipitation and CO_2_ uptake typically reach completion, and as a result, most experimental findings are based on observations made within this limited window of microbial activity. Most experiments investigating MICP do not consider the distinct growth phases of the microbes involved, such as the lag phase, exponential growth phase, or stationary phase.

This emphasis on the exponential phase has important implications for the interpretation and practical application of MICP. For instance, if one assumes that only the enzyme carbonic anhydrase is relevant and that its expression remains constant, then the number of enzyme molecules will increase exponentially alongside microbial growth. However, as nutrient availability declines and the culture transitions into the stationary phase, the number of enzyme molecules plateaus. At this point, the process likely achieves its maximum potential, but this peak is generally short-lived, and some form of media renewal will be required to maintain a healthy population of target microbes.

In a recent study by Seidel et al. (2025), a strain of *Sporosarcina silvestris* showed steady calcium carbonate precipitation during the stationary phase. While cell counts remained stable in that study, calcite precipitation continued over 24 h, demonstrating active mineralization beyond the exponential growth phase. To date, however, there has been limited research focused on the kinetics and biomineralization conditions necessary for efficient MICP beyond the initial growth phase. Even fewer studies (e.g., Murugan et al., 2021) have addressed strategies for maintaining maximum MICP performance in industrial systems over extended periods. Closing these knowledge gaps is essential for advancing MICP technology from controlled laboratory settings to practical, large-scale applications.

### Elevated CO_2_

In addition to these considerations, the effects of elevated CO_2_ on microbes and their gene expression have received only limited attention in most studies. While some studies suggest fewer profound impacts of elevated CO_2_ levels on microbial diversity (e.g., Ahrendt et al., 2014), the gene expression of key MICP enzymes under such conditions remains poorly understood (e.g., Xiao et al., 2015; Clarà Saracho and Marek, 2024). Indeed, Eklund (1984) showed that growth rates of MICP-capable bacteria like *Bacillus subtilis*, *Pseudomonas aeruginosa*, and *Bacillus cereus* were linearly inhibited with increasing CO_2_ concentrations by up to 40%.​ Elevated CO_2_ concentrations can have significant negative effects on microbial growth (Yu and Chen, 2019; Wan et al., 2018; Ennaciri et al., 2022) and, to some extent, on the gene expression of carbonic anhydrase (e.g., Xiao et al., 2015), presenting further scalability challenges for MICP. These adverse impacts are likely more pronounced in aerobic taxa, although concomitant acidification of the medium may also affect other microbial groups. Efforts to either maintain lower CO_2_ concentrations or employ taxa tolerant of elevated CO_2_ or pH instability, would be highly beneficial. In addition, use of anaerobes for MICP are largely unexplored, these organisms may have some advantages in settings where elevated CO_2_ atmospheres are being considered.

### Temperature

With a few exceptions (e.g., Okwadha and Jin, 2010; Omoregie et al., 2017; Peng and Liu, 2019; Zhang et al., 2021), temperature is often insufficiently addressed in MICP studies. Most research considers it only as necessary for supporting the growth of the target biomineralizing organism, despite its significant influence on both microbial activity and carbonate precipitation rates. Optimal MICP temperatures for many of the well-studied organisms, such as *Sporosarcina pasteurii*, are usually at relatively low temperatures of up to 30 °C (e.g., Omoregie et al., 2017). While elevated temperatures can accelerate reaction kinetics and enhance mineralization, enzyme activity lifespans may vary (e.g., Peng and Liu, 2019 and references therein). More research is thus warranted to explore organisms adapted to elevated temperatures. Furthermore, while these more efficient enzyme kinetics can be advantageous, they may require substantially higher process temperatures. This, in turn, necessitates thermotolerant microbial strains and greater energy input for heating, which undermines one of MICP’s main benefits, its potential for low energy demand and environmental sustainability. Further research is thus needed to explore this trade-off. If higher temperature processes are determined (through carbon accounting) to be preferable, work to develop or select robust, thermotolerant strains or enzymes will be crucial for advancing MICP technologies toward scalable and economically viable applications.

## Carbonate mineral stability

MICP produces a range of carbonate minerals of varying thermodynamic stabilities, most commonly calcite, aragonite, and vaterite (e.g., Anbu et al., 2016; Chang et al., 2017; Zehner et al., 2020), representing different carbonate mineral phases or polymorphs of the same chemical composition but different crystal structures. Metastable vaterite and aragonite are more susceptible to dissolution than calcite, but no single precise pH value universally defines the onset of dissolution for these minerals, since temperature, ionic strength and the degree of saturation of the solution also play a role (e.g., Cooke and Kepkay, 1980; Plummer and Busenberg, 1982; Svenskaya and Pallaeva, 2023 and references therein). Other phases important in MICP include amorphous carbonate as well as Mg-calcite (e.g., Défarge et al., 1994; Jones and Peng, 2014).

The optimal outcome for durable CO_2_ storage is the exclusive formation of calcite, the most stable and least soluble polymorph of calcium carbonate, which ensures long-term immobilization of carbon. However, achieving the exclusive formation of the calcite phase during MICP is a significant scientific and engineering challenge due to the complex interplay of biochemical and environmental parameters that govern carbonate polymorph selection. The MICP process can foster the nucleation of metastable phases such as vaterite and aragonite, which often appear either concurrently with or prior to calcite precipitation due to kinetic and local saturation effects (e.g., Khanjani et al., 2021). One core difficulty lies in the sensitivity of phase outcomes to subtle fluctuations in parameters such as pH, temperature, calcium and urea concentrations, bacterial species and activity levels, and the specific nature of additives or impurities present in the system (e.g., Dhami et al., 2013; Anbu et al., 2016 and references therein; Khanjani et al., 2021; Haystead et al., 2024). For example, high supersaturation or rapid mixing can promote the nucleation of vaterite or aragonite (e.g., Sun et al., 2015), while the presence of magnesium ions can stabilize these less stable forms and inhibit their transformation to calcite (e.g., Boon et al., 2020).

The cumulative effect of these interacting factors means that establishing a robust, reproducible method for suppressing metastable phases and reliably producing phase-pure calcite remains a persistent obstacle for both laboratory-scale and field-scale MICP applications. This challenge is further compounded by the inherent variability of biological systems and the natural progression of polymorphic transitions over time (e.g., Dhami et al., 2013; Khanjani et al., 2021).

Addressing these challenges through targeted process optimization and environmental monitoring will be essential. Placing a strong emphasis on developing and validating reproducible methods is paramount. Systematic approaches that include rigorous standardization of microbial inoculum, substrate concentrations, and environmental parameters, coupled with comprehensive reporting of experimental details, will significantly enhance cross-laboratory comparability. The adoption of reproducibility-focused protocols not only facilitates more reliable suppression of metastable phases but also enables consistent generation of phase-pure calcite, ultimately advancing the scalability and practical deployment of MICP technologies. This focus will help bridge current gaps between experimental success under controlled conditions and reliable performance in complex, real-world environments.

## Conclusion

While MICP holds potential as a biologically driven method for durable CO_2_ sequestration, realizing its full impact requires a more holistic and integrated evaluation. Current research focused on carbon sequestration often overlooks key factors such as whole-system carbon accounting, physiological constraints and the stability of the resulting carbonate minerals. Effective deployment of MICP must distinguish between transient organic biomass and stable mineral carbon, accurately track emissions from inputs, and account for microbial growth dynamics, carbon use efficiency, and tolerance to elevated CO_2_ and temperature. Additionally, reproducible control over polymorph formation, with an emphasis on generating stable calcite, is essential to ensure long-term carbon immobilization. Future progress depends on developing standardized methodologies, selecting robust microbial strains, optimizing nutrient sourcing, and validating mineral stability under field conditions. Addressing these challenges will be critical to transitioning MICP from a promising laboratory technique to a reliable and scalable solution for climate change mitigation.

## Data Availability

The original contributions presented in the study are included in the article/supplementary material, further inquiries can be directed to the corresponding author.
